# Subtle Increases in BMI within a Healthy Weight Range Still Reduce Womens Employment Chances in the Service Sector

**DOI:** 10.1371/journal.pone.0159659

**Published:** 2016-09-07

**Authors:** Dennis Nickson, Andrew R. Timming, Daniel Re, David I. Perrett

**Affiliations:** 1 Department of Human Resource Management, Strathclyde Business School, University of Strathclyde, Glasgow, Scotland, United Kingdom; 2 School of Management, University of St Andrews, St Andrews, Scotland, United Kingdom; 3 Department of Psychology, University of Toronto, Toronto, Canada; 4 School of Psychology and Neuroscience, University of St Andrews, St Andrews, Scotland, United Kingdom; University of Alabama at Birmingham, UNITED STATES

## Abstract

Using mixed design analysis of variance (ANOVA), this paper investigates the effects of a subtle simulated increase in adiposity on women’s employment chances in the service sector. Employing a unique simulation of altering individuals’ BMIs and the literature on “aesthetic labour”, the study suggests that, especially for women, being heavier, but still within a healthy BMI, deleteriously impacts on hireability ratings. The paper explores the gendered dimension of this prejudice by asking whether female employees at the upper end of a healthy BMI range are likely to be viewed more negatively than their overtly overweight male counterparts. The paper concludes by considering the implications of these findings.

## Introduction

Several studies have examined the effects of obesity and overweight on one’s chances of employment. Research has found that overweight job candidates suffered from bias in an experimental job interview [1]. The effect was particularly pronounced when the rater reported satisfaction with his or her own body image. Furthermore, *ceteris paribus*, “fat persons” are less highly rated on employability than normal-weight applicants [2]. Similarly, it has been found that obese job applicants were not only perceived to be less qualified than non-obese applicants, but were also less likely to be hired [3]. Consequently, excess weight is negatively associated with hireability ratings, among other work-related outcomes [4]. These findings are reflected in a recent meta-analysis [5], which points to adiposity as a major source of bias and prejudice [6] in the workplace. In short, there is already convincing evidence that *overt* obesity and overweight are linked to reduced employment chances.

Using mixed design analysis of variance (ANOVA), this study asks two questions that have not been explored in any depth, thus extending the above literature. First, to what extent does “subtle” but healthy weight gain reduce hireability ratings for female applicants against the backdrop of significant and unhealthy weight gain for their male counterparts? “Subtle” weight gain is defined here as a marginal increase in weight that is still within the normal and medically healthy BMI range. Second, do the effects of “subtle” weight gain vary for female job candidates applying for both customer-facing and non-customer-facing jobs in the service sector? To frame this second question, we employ the theory of aesthetic labour [7] in order to unpack the extent to which employee selection decisions are driven by customer expectations, which are anticipated to be harsher on women than men.

Overtly overweight and obese job applicants are less likely to be hired when compared to those who are of “normal” weight [5]. This effect is likely to be more pronounced when account is taken as to whether the job for which an adipose applicant is applying involves customer interaction [8]. Thus, it is argued that the obese are seen as being less suited for jobs that require interaction with customers, particularly when the organization places a strong emphasis on its image and appearance [4]. Similarly, Gruys [9] notes that “fat persons working in face-to-face sales environments are often assigned to nonvisible jobs” (p. 484). In this context, it is important to distinguish, geographically speaking, between jobs that are “front stage” and customer-facing, and those that are “backstage” and non-customer-facing, where the physical appearance of the employee is presumably of less significance to employers. As Goffman [10] notes, employers will often seek to hire those job applicants with “undesirable visual attributes for back region work,” whilst at the same time “placing persons who ‘make a good impression’ in the front regions” (p. 124).

Whether potential employees are seen to be appropriate for a “front stage” or “backstage” job will, to a large extent, be determined by their appearance and capacity for “aesthetic labour” [11] [12] [13]. The term aesthetic labour is analytically complex and a full working definition can be found in Nickson et al. [7]. Here, it is enough to note that firms employ people with certain capacities and attributes that favourably appeal to customers’ visual or aural senses, and which are sought through recruitment and selection processes and then developed through training and/or monitoring. Aesthetic labour recognizes how the capacities and attributes of employees can be conceptualized as “dispositions”, encompassing a range of aspects including, crucially for this study, the shape and size of the human body.

Williams and Connell [13] note how hiring managers often hire people with the “right look”, “to the exclusion of almost all other qualifications” (p. 353). As McDowell [14] corroborates, embodied attributes of workers become part of the service—“their height, weight, looks, attitude, are part of the exchange” (p. 9). Importantly, as MacDonald and Merrill [15] note in considering customer-facing service jobs, “race, gender, class and age coalesce in different job settings to create a norm of the worker who will ‘look the part’ given a particular service” (p. 123). A further key aspect of “looking the part” is concerned with customer perceptions around appropriate body shape, size and weight. For that reason, Gruys [9] suggests that “workers’ body size is an important trait to consider when examining aesthetic labor” (p. 484).

It is perhaps not surprising that, in considering workers’ body size and aesthetic labour, much of the extant research highlights that many service organizations are more likely to employ people for customer-facing jobs who are considered to be of “normal” weight or have a slim build [7]. Pettinger [11] found that “workers at [retail clothing shops] are not only fashionably dressed, they are young, *usually slim*, with ‘attractive’ faces” (p.178) (emphasis added). Similarly, Harris and Small’s [16] analysis of over 100 images in online promotional videos of hotel staff found that not one portrayed an overtly obese person; instead, 91 percent had a slim build, with only 9 percent being slightly larger, the majority of whom were men.

Within this context, a key theme of the emergent research on aesthetic labour is the importance of individuals’ appearance in the recruitment and selection process. It is the opportunity for organizations to filter in (and out) potential employees that they deem to be suitable (or unsuitable) for customer-facing positions [17] [18]. In considering recruitment and selection, there is evidence that employers often make decisions about potential employees based on first impressions. For example, Gatta [19] recounts how managers in a retail clothing shop recruited on the basis of “blink” decisions, prioritizing especially young, white, middle-class “girls” who would “look good” in the clothes sold in the shop. As she notes, the “blink moment” can often be a code for bias against certain characteristics that are deemed to be unsuitable for front line service work, including being adipose. Given the often visual cues that recruiters in service organizations use in selecting staff, appropriate corporeal attributes, including the shape and the size of the body, are, according to Huzell and Larsson [20], “an advantage, if not a pre-requisite, for employment in the service sector and for interactive service work in particular” (p. 105).

Scholars have also pointed to the gendered dimension of this “weight stigma”, such that women suffer significantly more prejudice than men when it comes to employment discrimination around their body shape and size [21] [22] [9] [16] [8]. This gendered dimension often reflects societal expectations of a particular type of female body [23], a point that is presumably more pronounced in customer-facing roles in the service sector [14] [11]. Where such an ideal body is not presented at the point of entry into an organization or maintained once employed, there is evidence of employment discrimination involving mostly customer-facing employees. For example, a number of recent cases in the US have offered support for the idea that employers can legitimately refuse to employ workers or fire existing ones who are considered overweight and who are working in customer-facing roles [24].

Given the foregoing discussion, this research makes an original contribution to the extant literature on weight-based discrimination by examining not only the extent to which slightly heavy—but not overtly overweight or obese—female job applicants face discrimination in employee selection, but also whether any discrimination varies by proximity to the customer. It throws light on the question of whether female job applicants on the upper end of the medically healthy BMI range suffer more bias than male job applicants who are overtly and medically overweight. It is on these points that the research makes an original contribution to extant debates. Thus, two overarching hypotheses are presented:

H1: A subtle simulated increase in adiposity for female applicants will have a significantly negative effect on hireability ratings.
H2: The negative effect of a subtle simulated increase in adiposity for female applicants is stronger in customer-facing roles and weaker in non-customer facing roles.

## Materials and Methods

### Stimuli

We initiated the experiment by drawing from a publicly available database of men’s and women’s faces (www.3d.sk). The images we selected for inclusion in the research were all taken under standardized lighting at the same distance. Faces were photographed from a 0˚ angle, with neutral expressions, hair pulled back and without any facial adornments. Inter-pupillary distance was also standardized. Both height and weight information for each subject was provided by the database. We settled on the faces of four men and four women (all Caucasians) for experimental testing. The eight faces spanned a range BMIs (female faces: mean BMI = 18.85 kg/m^2^, SD = 0.87, range: 17.81–19.95 kg/m^2^; male faces: mean BMI = 23.23, SD = 2.83, range: 20.90–27.36 kg/m^2^). Note that both averages are within a normal, healthy BMI range.

In addition to the eight original faces, we created a set of low- and high-BMI female and male “prototypes”. These prototypes were used as “models” to transform the original faces so as to make them appear “heavier”. We created four prototypes: low- and high-BMI female prototypes and low- and high-BMI male prototypes. The prototypes were created by digitally averaging ten faces of individuals with the desired BMIs [25]. The low-BMI female prototype had an average BMI of 17.85 kg/m^2^ (SD = 0.79). The high-BMI female prototype had an average BMI of 24.06 kg/m^2^ (SD = 6.37). The low-BMI male prototype had an average BMI of 22.19 kg/m^2^ (SD = 2.52), and the high-BMI male prototype had an average BMI of 26.47 kg/m^2^ (SD = 3.27). The faces used in the low- and high-BMI prototypes of each sex were matched for age (thus, an independent samples t-tests was conducted: both *t*(18) ≤ 0.39, both *p* ≥ .70). Matching prototypes for age was important in this case to ensure that the transformation of the original faces reflected shape changes that were associated with BMI differences, and not age differences.

**Fig 1:**
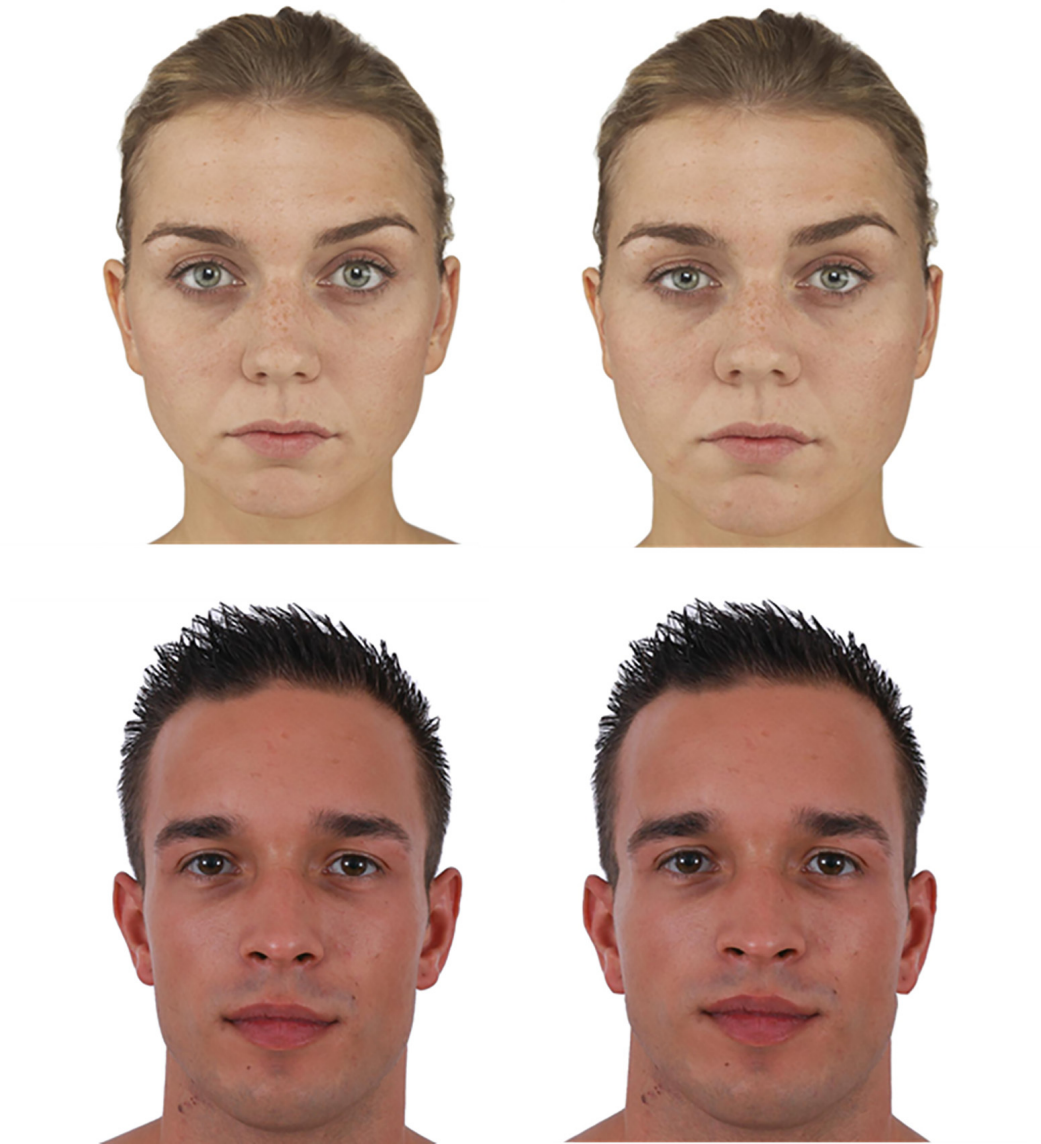
Original (left) and “heavy” (right) versions of test faces.

Using these prototypes as models, each of the eight original faces was then transformed to simulate an upward change in adiposity. To perform this transformation, we digitally-morphed each of the four test faces with the high-and low-BMI prototypes and transitioned the test face towards the shape of the high-BMI prototypes. We held the inter-pupillary distance constant to ensure that the faces did not simply grow larger overall as facial adiposity changed with the transformation. Because the BMIs of the original faces and the prototypes were known, we were able to use Psychomorph, a custom face-processing software [26], to transform each original face to the desired BMI values. This was achieved by applying the correct percentage of the face shape difference between the low- and high-BMI prototypes to each test face of the same sex. We transformed each test face to simulate an increase in BMI of 6.0 kg/m^2^, resulting in a total of 16 stimulus faces (4 men’s and 4 women’s original faces and their corresponding “heavier” versions). This method has been used in previous work to successfully alter perceptions of weight from faces [27]. Fig 1 provides an illustration of original and “heavier” versions of two of our experimental faces, one male and one female.

An additional 24 “diversion” faces were also introduced randomly among the original and “heavier” faces. These included faces that were manipulated by adding facial adornments (for example, tattoos or piercings) or by altering perceived race by changing the skin colour. We introduced these faces into the experiment in order to conceal from the respondents that this study was, in fact, “about” weight. The data from these “diversion” faces are not reported in this study since they are not relevant to its research questions. However, it is worth noting that the data on tattoos and piercings are reported in Timming et al [17], which employs the same sample as in the present study, but uses different variables from the dataset.

### Data Collection

In total, 121 women and 61 men completed the experiment over the course of 2013. The sampling frame from which the research participants were drawn included visitors to the University of St Andrews Perception Lab. All participants provided written consent, recorded through a digital consent form. The procedure was approved by the institutional ethics committee. In order to ensure the anonymity of participants, the only identifying data we collected from the sample were IP addresses. From the 182 valid respondents, we used a random number table to select 60 males (from 61 total male participants) and 60 females (from 121 total female participants) to be included in the sample (N = 120). The reason that we stratified the sample along the lines of participant sex was to promote homoscedasticity, thus complying with one of the assumptions of mixed design ANOVA. After stratification, we can report that the sample was: 50 percent female, 89.17 percent Caucasian and characterized by an average age of 25.67 years (*SD* = 10.47).

Although different social science disciplines espouse varying expectations regarding the appropriate size of a sample, in the specific field of facial perception research, a sample size of 120 participants is considered very large, especially in light of the fact that many studies in experimental psychology employ a much smaller number of cases in their samples [28].

The participants completed the experiment in an online laboratory. Each subject provided informed consent prior to completing the instrument. The experiment was carried out across two blocks: Block 1 and Block 2. Across both blocks, participants were instructed to assume that they are recruiters who need to hire someone from an applicant pool. In Block 1, the participants were asked to view all 16 test faces (along with the diversionary faces) and rate how likely they would be to hire each person for a *customer-facing job* (for example, a waiter or waitress, a receptionist or a sales assistant, etc.). In order to hold skills and work experience constant, participants were instructed to assume that all applicants were equally qualified for the position. Then they were asked to rate how likely they would be to hire each face depicted in the photographs on a 7-point scale (1 = extremely unlikely to 7 = extremely likely). The order of the presentation of faces was randomized in order to obviate the possibility of the respondents detecting a pattern.

After the participants completed Block 1, they were then asked to respond to Block 2. The same stratified sample was used for the analysis and the 16 faces (alongside the diversionary faces) were shown again to the participants in randomized order. In Block 2, the participants were instructed to assume that they are recruiting for a *non-customer-facing job* (for example, a back-of-house job like a chef, kitchen porter or stock assistant). For each of the 16 test faces, participants were asked to rate hireability on the same 7-point scale (1 = extremely unlikely to 7 = extremely likely). They were again instructed to assume that all job applicants were equally qualified for the position to which they were applying.

Inasmuch as we are interested in understanding the pure effects of the visual stimuli on hireability ratings, in both conditions, the participants were instructed to assume that all job applicants were equally qualified. Had we allowed qualifications to vary, we would then not have been able to attribute clearly the hypothesized reductions in hireability ratings to the subtle simulated increase in BMI evident in the faces.

### Analysis

The combined data from Block 1 and Block 2 are integrated into a single repeated-measures analysis in order to test statistically for the effect of job type (that is, customer-facing versus non-customer-facing roles) on the hireability of normal and subtly “heavier” female job applicants vis-à-vis overweight male job applicants. This integration is possible because the same participants completed both studies. Thus, statistical analyses were conducted in order to determine whether hireability ratings were affected by job type (*customer-facing*, *non-customer-facing*), sex of face (*male*, *female*), image type (*original version*, *“heavier” version*), and participant sex (*male*, *female*). On this basis, a 2x2x2x2 mixed design analysis of variance (ANOVA) was carried out, with participant sex entered as a between-subjects variable.

## Results

Table 1 reports the main effects of the experiment. The 2x2x2x2 mixed design ANOVA found a main effect of job type, with faces in the non-customer-facing jobs (M = 5.09, SE = 0.11) receiving higher employability ratings than those in the customer-facing jobs (M = 4.35, SE = 0.09; F(1, 118) = 75.81, *p* < .01, η_p_^2^ = .39). There was also a main effect of sex of face, with male faces (M = 5.04, SE = 0.09) receiving generally higher employability ratings than female faces (M = 4.40, SE = 0.10; F(1, 118) = 61.79, *p* < .01, η_p_^2^ = .34). There was another main effect of image type, with original versions (M = 4.84, SE = 0.09) receiving higher employability ratings than the corresponding “heavier” versions (M = 4.61, SE = 0.09, F(1, 118) = 36.00, *p* < .01, η_p_^2^ = .23). Interestingly, there was no between-subjects effect of participant sex (F(1, 118) = 0.16, *p* = .69, η_p_^2^ < .01), meaning that both the male and female participants rated the faces roughly equally.

**Table 1:** Summary of results of the 2x2x2x2 repeated-measures ANOVA.

	Effect type	Mean rating (SE)	Mean rating difference	F	*p*	η_p_^2^
**Job type**	Within-subjects	Non-customer-facing: 5.09 (0.11)	0.74	75.81	< .01	.39
(non-customer-facing; customer-facing)		Customer-facing: 4.35 (0.09)				
**Sex of face**	Within-subjects	Male: 5.04 (0.09)	0.64	61.79	< .01	.34
(male; female)		Female: 4.40 (0.10)				
**Image type**	Within-subjects	Original: 4.84 (0.09)	0.23	36.00	< .01	.23
(original version; “heavy” version)		“Heavy”: 4.61 (0.09)				
**Participant sex**	Between-subjects	Women: 4.76 (0.12)	0.07	0.16	.69	< .01
(men, women)		Men: 4.69 (0.12)				

**Fig 2:**
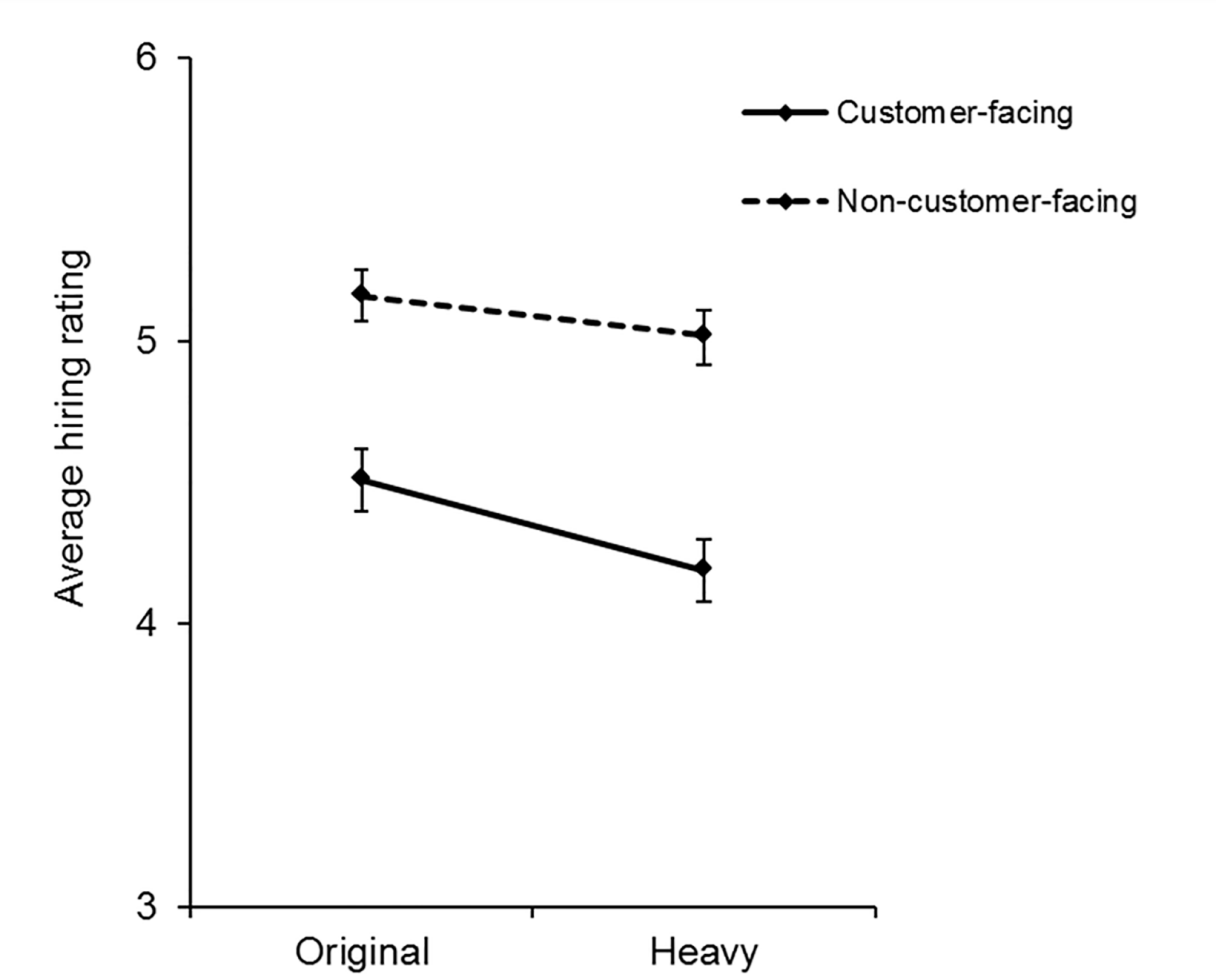
Average hiring ratings for non-customer-facing (dotted lines) and customer-facing (solid lines) jobs for the original and “heavy” versions of the testing faces with standard error bars. Original versions received higher ratings than “heavy” versions for both customer-facing and non-customer-facing jobs.

In addition to the 2x2x2x2 mixed design, we also tested for a series of relevant interaction effects, reported in Table 2. We found a significant interaction effect between job type and image type (F(1, 118) = 10.92, *p* < .01, η_p_^2^ = .09). Thus, we examined differences in hireability ratings between original and “heavier” versions separately for non-customer-facing and customer-facing jobs. A paired-samples t-test revealed that the difference in ratings between original and “heavier” versions was greater for customer-facing jobs (M = 0.32, SE = 0.05) than for non-customer facing jobs (M = 0.14, SE = 0.05; *t*(119) = 3.30, *p* < .01). Fig 2 illustrates graphically the relationship between the normal and “heavier” faces in customer-facing and non-customer-facing roles.

**Table 2:** Summary of the interactions between job type, sex of face, and image type and paired-samples t-test statistics.

Job type*image type			
	Mean difference (SE)—original minus “heavy”	*t*	*p*
Non-customer-facing	0.14 (0.05)	3.30	< .01
Customer-facing	0.32 (0.05)		
Job type*sex of face			
	**Mean difference (SE)—male minus female**	***t***	***p***
Non-customer-facing	0.53 (0.10)	2.49	.01
Customer-facing	0.74 (0.09)		
Sex of face*image type			
	**Mean difference (SE)—original minus “heavy”**	***t***	***p***
Female faces	0.66 (0.12)	3.08	< .01
Male faces	0.26 (0.08)		
Job type*sex of face*image type			
	**Mean difference (SE)—original minus “heavy”**	***t***	***p***
**Female faces**			
Non-customer-facing	0.18 (0.07)	3.29	< .01
Customer-facing	0.48 (0.08)		
**Male faces**			
Non-customer-facing	0.10 (0.06)	0.97	.34
Customer-facing	0.16 (0.05)		

There was also a significant interaction between job type and sex of face (F(1, 118) = 6.16, *p =* .01, η_p_^2^ = .05). Thus, we examined differences in hiring ratings between female and male faces separately for non-customer-facing and customer-facing jobs. A paired-samples t-test revealed that the difference in ratings between male and female faces was greater for customer-facing jobs (M = 0.74, SE = 0.09) than for non-customer facing jobs (M = 0.53, SE = 0.10; *t*(119) = 2.49, *p* = .01).

There was a significant interaction between sex of face and image type (F(1, 118) = 9.48, *p <* .01, η_p_^2^ = .07). Thus, we examined differences in hiring ratings between original and “heavier” versions, separately, for female and male faces. A paired-samples t-test revealed that the difference in ratings between the original and “heavier” versions was greater for female faces (M = 0.66, SE = 0.12) than for male faces (M = 0.26, SE = 0.08; *t*(119) = 3.08, *p <* .01).

The three way interaction between job type, sex of face and image type was marginally insignificant (F(1, 118) = 3.91, *p* = 0.05, η_p_^2^ = .03), which is not surprising given the reduced power of three-way interaction effects [29]. In order to militate against the possibility of Type II error, we examined the difference in ratings between the original and “heavier” versions of female and male faces, separately, for non-customer-facing and customer-facing jobs. We found that there was no significant difference between ratings of original and “heavier” male faces between non-customer-facing (M = 0.10, SE = .06) and customer-facing jobs (M = .16, SE = .05; *t*(119) = 0.97, *p* = .34). There was, however, a statistically significant difference between ratings of original and “heavier” female faces between non-customer-facing (M = 0.18, SE = .07) and customer-facing jobs (M = .48, SE = .08; *t*(119) = 3.29, *p* < .01). This finding suggests that the “heavier” women, even though they are still in a normal and healthy BMI range, are evaluated significantly more negatively in customer-facing roles than in non-customer-facing roles, but for men who are overtly overweight by BMI standards, there does not appear to be a significant difference across the two job types.

## Discussion

This research speaks to the challenges that women (and to a lesser extent men) face in what appears to be a highly “weight-conscious” labour market. We found that the marginally “heavier” female faces were rated lower on hireability than the original faces. Thus, a subtle simulated increase in one’s BMI, even within the healthy range, is a very real stigma that negatively impacts on women’s life chances [30]. Generally speaking, our “heavier” female faces were viewed more negatively than our “heavier” male faces, but our “heavier” female faces were also evaluated even more negatively in customer-facing jobs than non-customer-facing jobs, whereas for the “heavier” male faces that were overtly in the overweight BMI range, there was no statistically significant difference by job type. The main conclusion of this paper is that women within the normal BMI range appear to suffer greater weight-based bias than men who are overtly overweight. Although the focus of this paper is on women, it is also worth noting that a subtle simulated increase in adiposity also negatively impacted on the employability of men. Interestingly, the sex of the respondent was not significant in any of these evaluations. The implications of these findings are now discussed in turn.

### Research Implications

These results affirm that even a marginal increase in weight appears to have a negative impact on the hireability ratings of female job applicants. For women, it seems, even seemingly minute changes to the shape, size and weight of the body are important. As we have recognized, it is at the crucial point of recruitment and selection that organizations filter in (and out) potential employees that they deem to be appropriate (or inappropriate) for interactive customer-facing positions in particular. The present study points to evidence that organizations may potentially recruit on the basis of a perceived “fit” between the product and the employees’ embodiment of the product [7].

Inevitably, this finding gives rise to concerns about “lookism” [31]. It is important to recognize, however, that selecting female employees on the basis of their appearance is not, in and of itself, discriminatory, with Williams and Connell [13] noting that “branded service workers have little legal recourse today if they wish to challenge appearance rules in the workplace” (p. 351). Organizations may, then, circumscribe the appearance of employees if such regulations serve the purposes of branding and marketing of the organization and are in the business interests of the company. In other words, if companies can prove these points, they have a legal right to regulate employees’ image and appearance. Consequently, in the UK and US, “lookism”, *per se*, is not illegal, and it only becomes discriminatory if the basis of the discrimination is related to other protected characteristics, such as gender, ethnicity or disability [32]. Nevertheless, discrimination always has moral implications in respect to fairness [33].

This situation is unlikely to change any time soon, and again in both the UK and the US, weight is not a protected characteristic in fair employment laws [34]. So women in similar scenarios to those tested in our study would have no recourse to the law. What Hilpern [35] describes as “fatism” is, according to these results, seemingly alive and well because employers can legally deny women applicants a job based solely on appearance. The only time weight could become a potential area for discrimination is when it can be linked to disability [36] [37], but it is worth reiterating that the women in our study were still within the normal and healthy BMI range.

It is clear from our results that the gendered dimension of “fat stigma” is apparent in service employment at much lower BMIs than is currently recognized in the literature. Whereas previous researchers have confirmed that overtly obese and overweight women suffer from employment discrimination [22] [9] [16] [8], we show that even women on the high end of the medically normal BMI range are likely to suffer significantly more prejudice than even overweight men when seeking employment in the service sector. This point is further reinforced when account is taken of whether the job is customer-facing or not. Reflecting the point about societal expectations of a particular type of female body for customer-facing roles in the service sector [14] [11], this research concludes that “heavy but healthy” women were evaluated significantly less positively for such roles. This discrimination towards women can be compared to the findings in our research on men, where there was no significant difference between the two job types. Thus, when considering the impact of adiposity across customer-facing and non-customer facing jobs, our research supports the view that heavier women are not only less likely to secure a job, but also, if they do secure a job, more likely to be assigned to non-visible, backstage roles, as opposed to front stage, face-to-face roles [9] [16].

### Practical Implications

This research has clear implications for those responsible for hiring employees in the service sector, and also for job seekers who are seeking employment. For those responsible for hiring employees, it would seem important for them to recognize the potential for weight-based bias against applicants within the recruitment and selection process, even though such recognition may not necessarily change their views about such applicants. Thus, although Finkelstein et al [4] suggest that “certain industries may have to be more vigilant in protecting against potential weight bias than others” (p. 219), in reality, it is these very industries, such as retail and hospitality, that are often reliant on projecting a certain image to customers and that are more likely to select employees whom, they feel, appropriately “fit” the brand image [7] [13]. Consequently, some employers may argue that they are making a logical and rational decision in terms of whether slightly heavier women, as opposed to slimmer ones, would be appropriate for customer-facing jobs. This point is acknowledged by Larkin and Pines [2]:

A normal weight preference in hiring might not be viewed as discriminatory at all; an employer might very well feel that he could easily justify (even to himself) hiring a normal weight person in preference to an objectively overweight applicant … Hiring the overweight might be bad for business since the potential clients or customers either find the overweight person distasteful or associate the condition with deficient performance (p. 325).

Consequently, whilst the ethical impulse in us might suggest that weight should not be an influential factor in recruiting employees, clearly our data, and other studies reported in the paper, demonstrate that, within the workplace, and particularly the service workplace, discrimination against women, even those on the upper end of the normal BMI range, remains real, current and widespread.

In recognizing the extent of weight-based discrimination against applicants, Finkelstein et al [4] advocate the need for sensitivity training for those recruiting in order that they do not actively discriminate. As noted above, though, this appeal to employers to act ethically may not necessarily find success. A further argument could be made in terms of the business case and ensuring appropriate workplace diversity to reflect customer diversity. The number of people who are overweight or obese is continuing to grow at a significant rate [38] and it could be argued that companies should recruit employees who are representative of the general population. In this way, the case could be made that service firms should have a suitably diverse workforce reflecting their customer base. For example, the average size of women in both the UK and US is equivalent to a BMI of around 27.5 units [39], so it would seem important that the workforce should reflect this point. Moreover, within the specific context of the hospitality and retail industries it is argued that employers can seek to educate both co-workers and customers by seeking to promote positive images of capable and competent heavy employees through presenting counter-stereotypic information to counter discrimination and stigmatization [40] [8].

For jobseekers, the evidence might seem to suggest that they need a certain amount of pragmatism if they are seeking a customer-facing job in the interactive services sector, but from an ethical standpoint, we cannot advocate this conclusion. It would be fundamentally wrong to suggest that women (or indeed men) of any size—whether a medically healthy BMI, overweight or obese—should not seek a customer-facing job because of their appearance. Powerful societal standards of beauty should be actively worked against, rather than just accepted as a matter of fact. One of the best ways of countering these standards of beauty is to publicize studies such as this one that shine a light upon the unrealistic challenges that women face in a highly weight-conscious labour market and to draw much needed attention to the fact that the women respondents in our study were equally likely to rate “heavy but healthy” female job applicants just as negatively as the male respondents.

### Study Design Limitations

This research is susceptible to the same set of critiques that are levelled against all studies in experimental psychology. The key challenge in laboratory-based studies is to approximate, as best as possible, “real life” conditions, but it is not possible to re-create them exactly. For example, in the real world, skills and qualifications are never held constant like they are in the present study. Furthermore, our respondents are analogues for hiring managers since they are role-playing. This is not, in itself, a devastating critique since analogue studies are widely accepted in experimental psychology. Furthermore, a now classic study by Bernstein et al. [41] verified, via extensive validation checks, that employee selection decisions made by analogues are roughly “comparable” to those of practicing hiring managers in any case. But it is still worth considering the value of lab-based studies vis-à-vis field studies.

Dipboye [42] has suggested that, “in the debate over alternative research strategies and settings, the problems of the laboratory are exaggerated, whereas many of the problems of field research are de-emphasized or completed ignored” (p. 25). He rightly concludes that lab and field studies complement one another, with each addressing the other’s limitations. Falk and Heckman [43] recently criticized those who argue that lab experiments lack generalizability. They argue that the real value of experiments lies in their ability to establish internal, as opposed to external, validity. So, whilst we recognize the limitations of this research design, it never makes sense to throw out the proverbial baby with the bathwater. The key advantage of this experimental design is that it affords us the ability to hold factors constant and standardize the stimuli in a way that would be impossible in the field.

Another limitation that deserves mention relates to the non-random nature of the sample. The fact that it was composed in an online laboratory is not, in itself, a serious problem since it has already been proved that the results of web-based studies such as this one are generally comparable to those derived from the more traditional sampling methods [44]. This limitation should be recognized, but is also tempered by the fact that, as established above, we are much less concerned with the generalizability of our findings than with establishing internal validity.

It is also important to acknowledge that there may be concerns as to whether the study is capturing what it seeks to—the phenomenon of how slight increases in BMI affect hireability. For example, there may be an argument to suggest that this this study is, in fact, capturing differences in facial attractiveness rather than BMI. We would seek to allay these concerns to an extent by pointing out the work of Agerström and Rooth and Rooth [45] [46] which similarly uses facial photo manipulation to assess hiring discrimination against obese individuals, whilst also accepting potential limitations in such an approach. As Agerström and Rooth [45] acknowledge, “by choosing to manipulate photos of normal-weight applicants into looking obese, we also changed their physical attractiveness. Thus, it is possible that the hiring managers’ decisions reflected discrimination against unattractiveness” (p. 800). Consequently, they advocate continuing research which seeks to further examine the issue of obesity and attractiveness and how they may be intertwined, a call which we would equally endorse. In a similar vein, when considering issues of obesity and attractiveness, there may be different outcomes in different service occupations and this is an area worthy of further research. For example, in some front stage occupations, such as a cashier, the majority of the employees’ bodies may be obscured from the view of customers, whilst for others, such as waiters, they are seen fully from all angles and not standing behind a barrier. The recent decision in the “Borgata Babes” case provides an extreme example of how organizational requirements around weight and appearance will be particularly stringent in occupations such as being a cocktail server in a casino [24], though again this remains an area worthy of further research to see how this issue varies in other occupational settings.

Finally, it is worth noting that these results are limited to the experiences of white job applicants in the labour market. Holding the race constant in the test faces was important given that the use of multiple races in the experimental line-up could have resulted in biased ratings that were based on in-group favouritism and stereotypes [47]. But in spite of this justification for using only Caucasian faces, the lack of racial and ethnic diversity is still a limitation of the present study. To be sure, further research using non-white job applicant faces is needed in order to establish the robustness of the findings and to better understand how the intersectionalities [48] of weight and race impact on employability.

## Conclusion

The results of this experiment are deeply unsettling from the viewpoint of gender equality in the workplace. It was found that, in service sector employment, women on the upper end of a normal and medically healthy BMI range face greater weight-based prejudice than men who are clearly and overtly overweight. In behind-the-scenes jobs in particular, the “heavy but healthy” women were rated lower on employability than the slimmer women, but there was no statistically significant difference between the overtly overweight men and their original faces. In short, a little weight gain for female job applicants is damaging to women’s job chances. These findings suggest quite clearly that women are at a distinct disadvantage compared to men in relation to their “gendered physical capital” [49].
